# ESR Essentials: diagnosis and assessment of treatment response in patients with luminal Crohn’s disease—practice recommendations by the European Society of Gastrointestinal and Abdominal Radiology

**DOI:** 10.1007/s00330-025-11620-2

**Published:** 2025-06-11

**Authors:** Maira Hameed, Isabelle De Kock, Jaap Stoker, Stuart A. Taylor

**Affiliations:** 1 Department of Radiology, University College London Hospitals, London, UK; 2 Centre for Medical Imaging, Division of Medicine, University College London, London, UK; 3 Department of Radiology, Ghent University Hospital, Ghent, Belgium; 4 Department of Radiology and Nuclear Medicine, Amsterdam UMC, University of Amsterdam, Amsterdam, The Netherlands; 5 Amsterdam Gastroenterology Endocrinology Metabolism, Amsterdam, The Netherlands

**Keywords:** Crohn’s disease, Magnetic resonance imaging, Ultrasound, Intestine, Small bowel

## Abstract

**Abstract:**

The European Society of Gastrointestinal and Abdominal Radiology (ESGAR) presents an ESR Essentials review article on Crohn’s disease (CD) diagnosis and treatment response assessment. The focus is on luminal disease, particularly in the small bowel, and to a lesser degree, the colon. Magnetic Resonance Enterography (MRE) and ultrasound are typically the first-line radiological investigations for known or suspected luminal CD. They are accurate for assessing the entirety of the bowel wall and extra-enteric tissues and are generally well tolerated by patients. CT has utility as well, especially in the acute setting. Disease location, extent, and phenotype should be assessed. Well-validated imaging signs of acute inflammation (active disease) are responsive to therapeutic interventions and include bowel mural thickening and oedema, perimural inflammation, ulceration, and hypervascularity. Other phenotypes (stricturing or penetrating with fistulae and/or abscesses) can coexist, and the predominant disease phenotype should be established. We recommend that radiologists work closely within multidisciplinary teams to optimise imaging selection for individual patients, tailored to the clinical question. Findings should be clearly communicated to best inform management decisions using standardised terminology, and structured reporting of disease status, treatment response categorisation, and any associated complications.

**Key Points:**

- *MR Enterography and intestinal ultrasound are the key imaging modalities for the diagnosis and follow-up of luminal CD, with CT reserved primarily for the acute setting*.
- *Inflammation, or active disease, is the hallmark of luminal CD, and there are several validated mural and extramural imaging signs of activity, often coexisting with chronic changes*.
- *Changes in the imaging markers of active luminal CD are central to radiologists' judgement of treatment response and should be communicated in a standardised report in addition to any stricturing or penetrating complications*.

## Key recommendations

- Cross-sectional imaging has a central role in luminal Crohn’s disease diagnosis and follow-up, including assessment of treatment response and complications. MR Enterography and intestinal ultrasound (IUS) are accurate and well tolerated. CT is typically used as a second line or in the acute setting (level of evidence: high).
- There are several validated mural and extramural signs of inflammation (active disease) which should be assessed; bowel mural thickening, mural and perimural oedema, ulceration, and hypervascularity. They are frequently superimposed on chronic disease (chronic inflammation, fibrotic, fat, and smooth muscle changes) in the same bowel segment, manifesting as mural thickening, but also mural fat deposition, fibrosis, pseudosacculation, and fibro-fatty proliferation. A judgement should be made of the predominant phenotype to aid appropriate management (level of evidence: high).
- The imaging signs of active disease are also responsive and used in the assessment of treatment response which should be expressed according to the European Crohn’s and Colitis organisation (ECCO)-ESGAR recommendations; (i) transmural remission, (ii) significant transmural response, (iii) stable disease, and (iv) progressive disease with or without stricturing or penetrating complications. To aid clear communication within the multidisciplinary team, structured reports using standardised terminology are recommended (level of evidence: high).

## Introduction

Crohn’s disease (CD) is a lifelong relapsing and remitting condition affecting any part of the gastrointestinal tract, characterised by patchy transmural inflammation. Patient symptoms do not always accurately reflect disease inflammatory burden, and clinicians need objective information on the extent and activity of the disease and presence of (extra)mural complications to optimise patient management. Although endoscopy is still the reference standard for CD activity assessment, its limitations are increasingly recognised. Less invasive, less expensive, and better tolerated cross-sectional imaging techniques (US, CT, and MRI) have emerged as viable alternatives to endoscopy for both diagnosing and monitoring CD [1, 2]. Moreover, cross-sectional imaging modalities have distinct advantages over endoscopy as they allow visualisation of the full length of the small bowel beyond the reach of endoscopy and enable assessment of the full thickness of the bowel wall (transmural evaluation), deep to the endoscopically visualised mucosa, as well as peri-enteric evaluation. The management strategy for CD has shifted from controlling symptoms alone to a proactive “treat-to-target” approach, which involves regular assessment of disease activity based on objective outcome measures with the aim of achieving disease control and avoiding long-term complications [3]. Cross-sectional imaging plays a crucial role in this treat-to-target strategy.

This ESR essentials review from ESGAR (European Society of Gastrointestinal and Abdominal Radiology) provides a comprehensive overview of the high-level evidence around the use of cross-sectional imaging for diagnosis and treatment response assessment in luminal CD with practical guidance for daily clinical reporting, imaging selection, and communication within the multidisciplinary team.

## Diagnosis

The pathogenesis of CD is not yet fully understood but is thought to involve a complex interchange between genetic disposition, environmental factors, immune factors, and the gut microbiome. CD can affect any part of the gastrointestinal tract, but most commonly, the terminal ileum is involved. The main symptoms are abdominal pain, diarrhoea, fatigue, and weight loss, although the clinical presentation varies widely. Other conditions may mimic CD, such as intestinal infections (e.g., *Yersinia enterocolitica*, *Clostridium difficile*, *Mycobacterium tuberculosis*), connective tissue disorders, vasculitis, ischaemia, eosinophilic enteritis, non-steroidal anti-inflammatory drug use, and neoplasms. The diagnosis of CD, therefore, involves a combination of clinical, biochemical, radiological, endoscopic, and histological investigations [1].

The behaviour of the disease has been classified into three phenotypic subtypes based on the degree of transmural involvement, although there is ongoing debate as to whether current classification systems should be further refined to reflect the dynamism of the disease and overlapping subtypes [4, 5]. (1) The inflammatory subtype is characterised by acute inflammation of the gastrointestinal tract with no fistulising or stricturing complications. (2) The stricturing subtype presents with chronic changes (chronic inflammation with varying levels of fibrosis, smooth muscle thickening, and fat deposition) and luminal narrowing with or without obstruction. (3) The penetrating subtype manifests by the development of transmural complications, such as fistulae or abscesses. In addition to these subtypes, patients can develop perianal disease (fistula and/or abscess), which can occur regardless of the underlying luminal disease phenotype [4].

Timely diagnosis, accurate phenotyping, and staging of CD are essential for optimising patient management, with a key role for cross-sectional imaging. MR Enterography (MRE) and IUS have emerged as the primary cross-sectional small bowel investigations, as both are accurate in the initial diagnosis and ongoing assessment of CD [6]. The METRIC study group reported that both MRE and IUS had a high sensitivity and specificity for detecting active small bowel disease (97% and 96% for MRE; 92% and 84% for IUS [6]. However, MRE was superior in assessing the extent of disease in the small bowel, particularly in ileal disease upstream of the terminal ileum, with a sensitivity of 80% and a specificity of 95% for MRE, significantly higher than IUS (70% and 81%, respectively). Therefore, when available, MRE is usually preferred over IUS for accurate staging at the time of diagnosis when the disease distribution and phenotype are first defined. IUS is sensitive for detecting CD in the small bowel, so it is a useful first-line investigation for initial diagnosis. Due to ionising radiation exposure, CT is largely reserved for the emergency setting or when non-ionising alternatives are not accessible. The decision regarding which cross-sectional technique to employ is discussed below. Recommended MRE, CT, and IUS imaging acquisition protocols are listed in Table 1.

**Table 1:** Image acquisition protocols for IUS, CT, and MR enterography (MRE)

Ultrasound	MR	CT
		
RECOMMENDED SCANNING PROTOCOL		
Initial systematic scan	Minimum sequences	
• Convex probe (low frequency 3.5–5 MHz)	• Coronal SSFPGE without FS • Axial and coronal T2 FSE without FS • Axial or coronal T2 FSE with FS	• Abdomino-pelvic scan after IV contrast administration during enteric phase (45 s) or portal venous phase (70 s) • Multiplanar reformats
Detailed bowel scan	Optional sequences (add 2 of 3)	
• Linear probe • (high frequency 6–11 MHz^*^) • Colour Doppler to assess vascularisation of abnormal segments, low flow range (5–7 cm/s)	• Axial and coronal pre- and post- contrast 3D T1-weighted GE with FS (60–70 s) • Axial DWI (*b*-values 50 and 600) • Coronal cine balanced SSFP	

*SSFPGE* steady-state free precession gradient echo, *FSE* fast spin echo, *FS* fat saturation, *3D* 3-dimensional, *GE* gradient echo, *DWI* diffusion-weighted imaging, *SSFP* steady-state free precession

* Higher frequency probes can be used if available

## Disease activity assessment

### Imaging findings of active disease

**Fig. 1:**
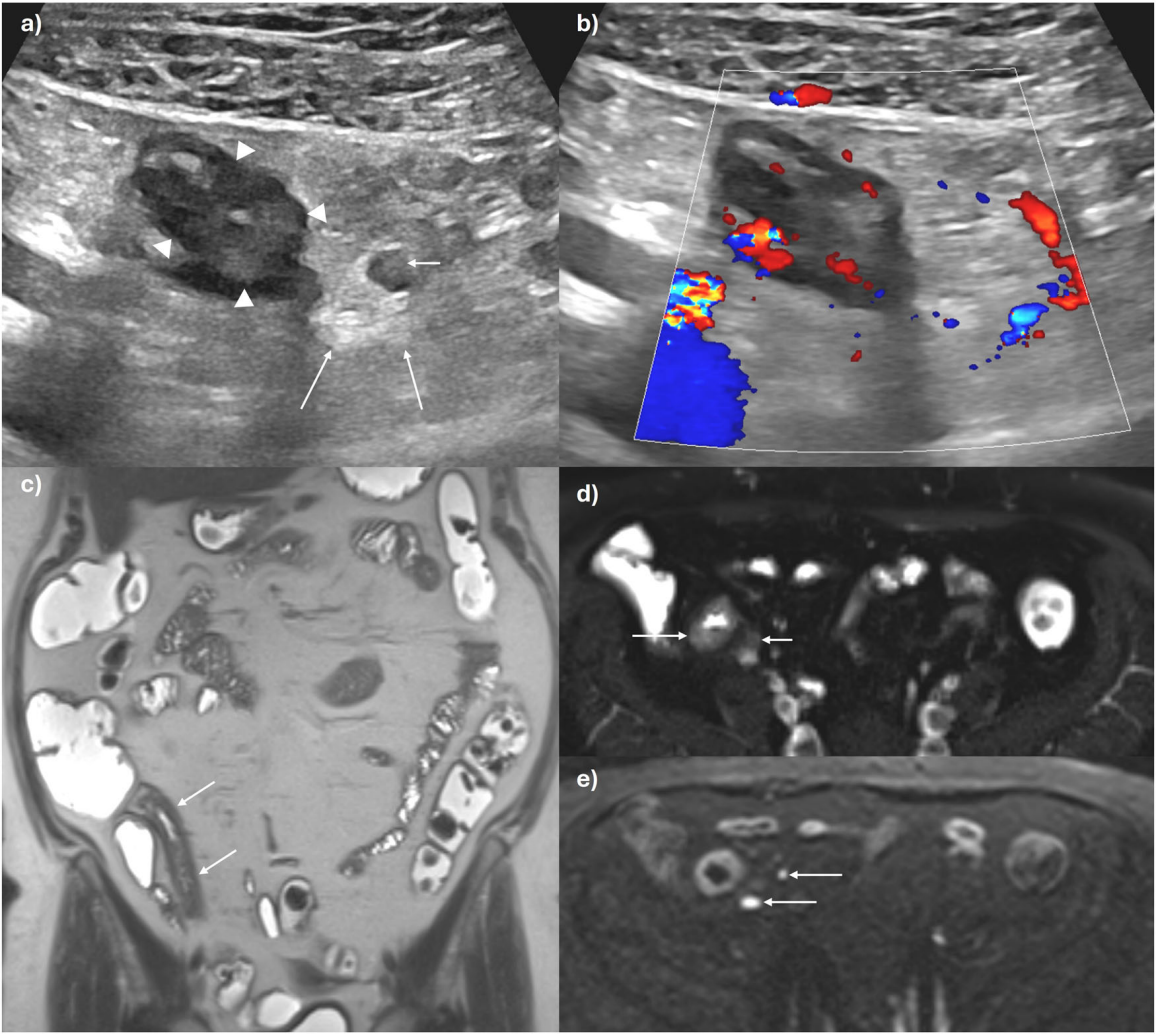
A 59-year-old female with a new diagnosis of CD. **a** IUS shows a thickened terminal ileum with extensive loss of mural stratification (arrowheads), hyperechoic mesenteric fat (long arrows), and an enlarged mesenteric lymph node (short arrow). **b** There is increased vascularity in the bowel wall on colour Doppler extending into the inflammatory fat. **c** Coronal T2-weighted image again shows the thickened terminal ileum (arrows). **d** Mural oedema (long arrow) and perimural inflammation (short arrow) in the terminal ileum are clearly depicted on this axial T2-weighted image with fat saturation. **e** Axial diffusion-weighted image (b600) demonstrates restriction in the inflamed terminal ileum with adjacent lymph nodes (arrows)

**Fig. 2:**
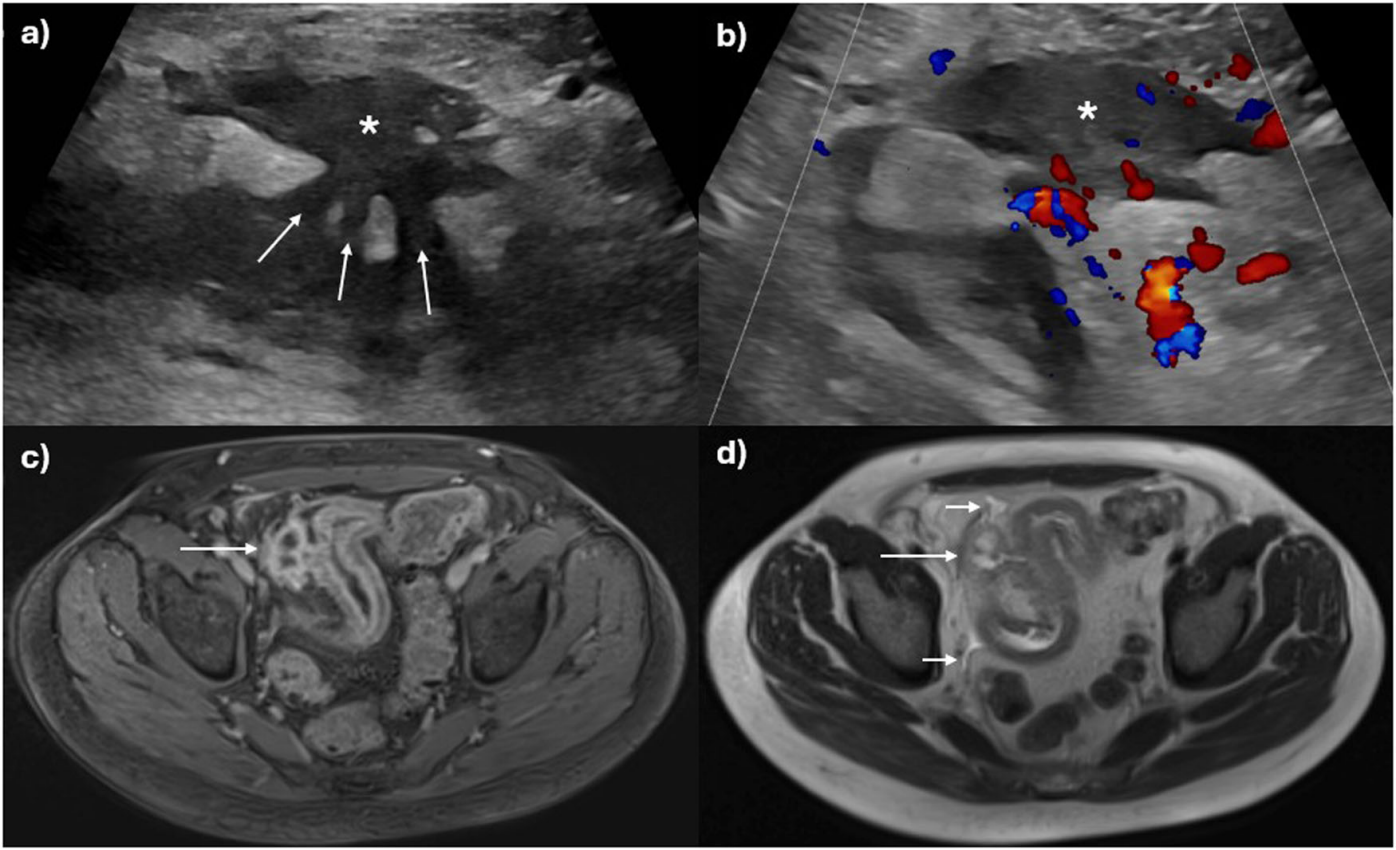
A 32-year-old male patient presents with right lower quadrant pain and elevated inflammatory blood markers following ileocaecal resection. **a** Ultrasound reveals several fistulous tracts (arrows) originating from the inflamed neoterminal ileum, converging into a hypoechoic inflammatory mass (asterisk) with internal vascularisation on colour Doppler imaging (**b**). A 41-year-old male patient with worsening symptoms and high inflammatory blood markers. **c** MR Enterography shows active disease in the distal ileum with layered, mural hyperenhancement on the axial fat-saturated T1-weighted post-contrast image, along with a rim-enhancing abscess (long arrow). **d** Axial T2-weighted image depicts small pockets of free fluid (short arrows) near the inflamed distal ileum and adjacent abscess (long arrow)

Consensus guidelines, including from ESGAR, recommend standardised nomenclature for interpreting and reporting cross-sectional imaging in CD patients, to improve communication between teams and increase reporting consistency [7, 8]. Several radiological features of active disease (inflammation) have been validated against endoscopy, histopathology, and biomarkers in the blood and stool. The main imaging findings associated with inflammation in CD include, bowel mural thickening, mural oedema, perimural inflammation, ulceration, and increased vascularity (Table 2) [9–13]. Bowel mural thickening is the most robust parameter for evaluating disease activity, although nearly all pathological bowel conditions can cause thickening. A threshold of > 3 mm measured in a well-distended bowel segment is generally accepted as a sign of active disease in both the small bowel and colon (Figs. 1 and 2). However, as there is significant overlap between acute inflammatory and chronic changes in diseased bowel in CD, other activity parameters should also be assessed. Chronic disease (chronic inflammation, fibrotic, fat and smooth muscle changes) can indeed manifest as mural thickening along with mural fat deposition, fibrosis, pseudosacculation, and fibro-fatty proliferation. On IUS, mural oedema may cause disruption of the normal 5-layer bowel mural stratification, particularly patchy low echogenicity within thickened submucosa. On MRE, increased mural T2 signal is a highly specific indicator of disease activity, typically reflecting moderate to severe inflammation (Fig. 1). High signal intensity on non-fat saturated T2-images can also correspond to intramural fat due to prior inflammation, but can be differentiated from oedema using fat-suppressed T2 sequences. Similarly, increased T2 signal and stranding of the mesenteric fat due to perimural inflammation generally represent active disease in the context of CD-affected bowel and are best appreciated on fat-saturated T2 images. On IUS, inflammatory fat is hyperechoic and may also show increased colour Doppler signal (Fig. 1). Another marker of disease activity, deep ulceration, can be seen on both IUS and MRE as focal mural defects in the intraluminal surface, with mural extension of air or intraluminal oral contrast, respectively. Increased vascularisation and neoangiogenesis in inflammatory bowel segments are reflected by mural hyperenhancement and engorged vasa recta (“comb sign”) on intravenous contrast-enhanced MRE images, and increased mural and extra-mural signal in and around inflamed bowel on colour Doppler IUS (Figs. 1 and 2). Additional imaging findings, such as diminished motility, may also reflect intestinal inflammation [9–11]. Abnormal small bowel loops show reduced peristalsis compared to normal small bowel. IUS enables real-time depiction of altered peristalsis, showing rigid, thick-walled small bowel segments. Cine-MRI techniques can detect the same findings but also allow quantification of small bowel motility using dedicated software. A multicentre, prospective study showed that a reduction in terminal ileal motility measured by MRE correlates with histopathological and endoscopic disease activity [14]. Finally, the presence of enlarged mesenteric lymph nodes, although nonspecific, may also indicate active disease. Diffusion-weighted imaging (DWI) is an additional MRI method that can provide information on tissue composition and histology. High DWI signal on high *b*-value images with corresponding low signal on the apparent diffusion coefficient (ADC) map (restricted diffusion) is usually seen in bowel affected by IBD, reflecting hypercellular tissue. While DWI is useful for detecting active disease, it cannot be used exclusively, as fibrosis also causes restricted diffusion [9, 11]. Of note, in mild disease, cross-sectional imaging may not show any abnormality, particularly as superficial ulceration is often not visible. IUS usually provides a more detailed visualisation of the mucosa than MRE, however, currently, there is no data confirming that IUS is superior to MRE in detecting mild inflammation.

**Table 2:** Main imaging findings on MR Enterography (MRE) and IUS associated with active disease (i.e., inflammation) in CD

Imaging findings	Description/definition		Illustration	
	MRE	IUS	MRE	IUS
Thickened bowel wall	> 3 mm bowel wall thickness			
Mural oedema	High mural T2 signal, best seen on fat-suppressed T2 sequences (arrows)	Focal or extensive loss of mural stratification (arrowheads)		
Perimural inflammation	High T2 signal in mesenteric fat (arrows) adjacent to the inflamed bowel segment, best seen on fat-suppressed T2 sequences	Hyperechoic fat (arrows) and/or free fluid adjacent to the inflamed bowel segment		
Ulceration	Focal defect in the intraluminal surface of the bowel wall (arrows) with extension of intraluminal contents or air into the bowel wall			
Increased vascularisation	Mural hyperenhancement (arrows), engorged vasa recta (“Comb sign”) (arrowheads)	Increased (extra-) mural colour Doppler signal		

### Activity scores

A variety of MRE and IUS activity scores have been developed and internally and externally validated against a range of reference standards, with high interrater reliability reported [9–12]. The first and best validated MRE score is the magnetic resonance index of activity (MaRIA), encompassing bowel mural thickening, mural oedema, ulceration, and mural contrast enhancement. Other MRE-based indices such as the simplified MaRIA (sMaRIA), London, ‘extended’ London, and Clermont scores generally consist of similar parameters with differences in the need for intravenous contrast administration or DWI. Most of the IUS activity scores focus on bowel mural thickness, disrupted mural stratification, increased colour Doppler signal, and inflammatory fat. Examples include the Bowel Ultrasound Segmental Activity Score (IBUS-SAS), Bowel Ultrasound Score (BUSS), and Simple Ultrasound Score for Crohn’s Disease (SUS-CD). Although activity scores are primarily used in research and clinical trial settings, given their relative complexity and the length of time required for their calculation, their incorporation into routine clinical reporting is encouraged, as they help make the assessment of imaging findings more objective and systematic.

## Treatment response assessment

The overall aim of CD management is to target and treat inflammation, i.e., active disease, to avoid irreversible bowel damage and adverse long-term outcomes [3, 15, 16]. There are a growing number of drugs, most prominently biologic agents, used in this aggressive “treat-to-target” approach. MRE and IUS are also central in the treatment response setting and well tolerated by patients who require lifelong monitoring [9, 17, 18]. Only cross-sectional imaging can detect healing of the full thickness of the bowel wall (transmural healing), although subtle mucosal disease, only visible on capsule endoscopy, could be missed. In clinical practice, most patients are followed up using cross-sectional imaging, with capsule endoscopy reserved for problem-solving in a very limited number of cases. The improved prognostic value of achieving this treatment target of transmural healing over endoscopic mucosal healing alone is increasingly recognised [19]. A crucial element of the radiologist's assessment during follow-up is to clearly communicate the degree of treatment response, including any residual untreated inflammation that may be targeted, and the presence of any complications [7].

### How to classify treatment response

ECCO-ESGAR recommendations describe four main categories of treatment response [7].

(1) Transmural remission or healing. As noted, patients achieving healing of the whole bowel wall have improved long-term outcomes compared with those achieving endoscopic mucosal healing alone [20]. However, transmural healing is only achieved by around 25% of patients at up to 1 year using a strict definition of normalisation of the bowel wall, i.e., all markers of mural and extramural disease activity are normalised, including bowel mural thickness ≤ 3 mm [21, 22]; (2) Significant transmural response (unequivocal improvement in activity parameters and/or extent), (3) Stable disease (no appreciable change), and (4) Progressive disease (unequivocal worsening of activity parameters, new disease sites and/or new complications).

There are currently no strict, objective criteria for response groups 2 to 4, reflecting that disease activity scoring systems are rarely applied in routine clinical practice.

**Fig. 3:**
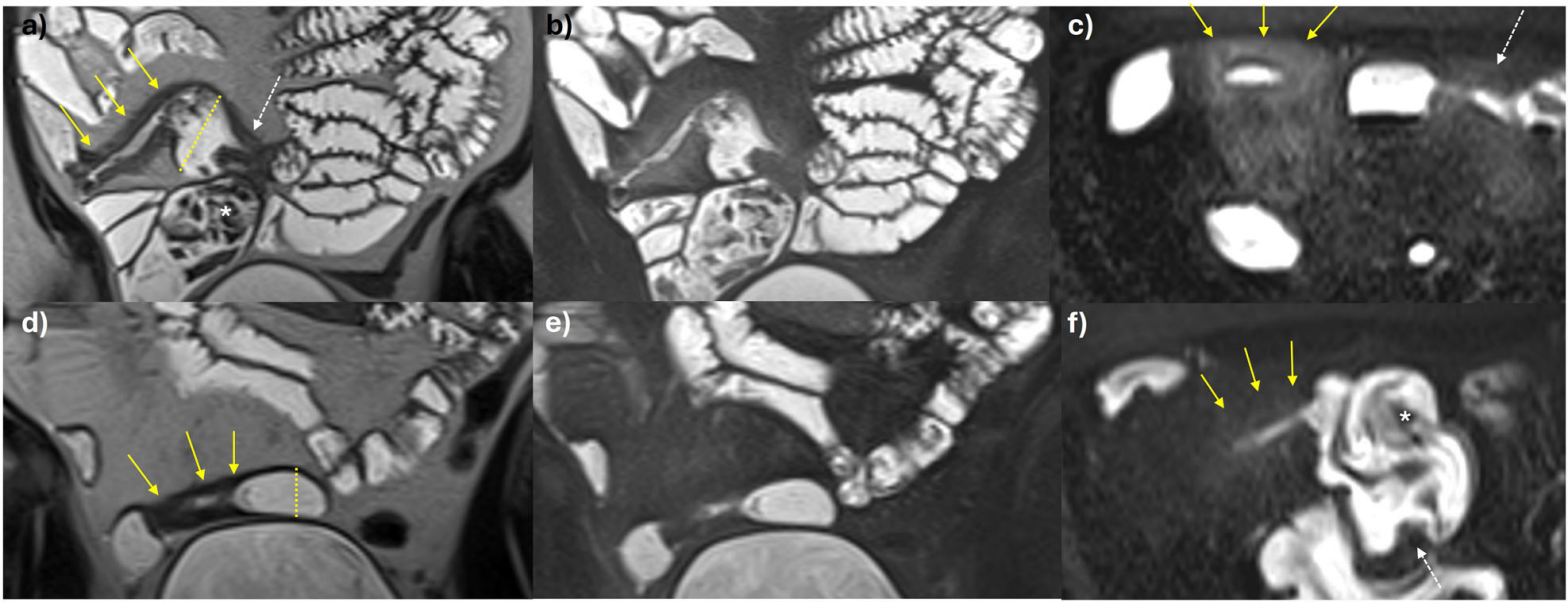
Treatment response assessment. 34-year-old female patient who stopped previous biologic treatment due to developing antibodies and presented with worsening diarrhoea. Baseline MRE study: **a** coronal T2-weighted image shows distal ileal disease with mural thickening (solid arrows) and perimural fat stranding with dilation of the immediately upstream loop of bowel (dashed line), indicating active, stricturing disease. Above this, there is a shorter stricture (dashed arrow) with upstream bowel dilation with enteroliths (asterisk). **b** Coronal and (**c**) axial T2 fat-saturated images show corresponding increased mural and perimural signal reflecting oedema in these same segments (solid and dashed arrows). The patient started Ustekinumab treatment, and a repeat MRE was performed 6 months after baseline to assess for response. **d** Coronal T2-weighted, (**e**) coronal, and (**f**) axial T2 fat-saturated images show significant transmural response. There is reduced mural and perimural T2 signal (oedema) shown in the longer segment of disease (arrows) and improved upstream dilation (dashed line). Although activity in the shorter segment of stricturing disease (dashed arrow) has improved, there remains proximal dilation mostly due to chronic disease (asterisk). MRE, MR enterography

Radiologist classification is by subjective assessment of the aforementioned validated disease activity parameters at baseline and follow up; bowel mural thickening, mural and perimural oedema, hypervascularity, ulceration, and, in IUS, bowel mural stratification (Fig. 3). These parameters are generally responsive to therapeutic changes and concordant with endoscopic findings [16]. Other signs related to disease activity can also be used to help judge response, such as improvements in enlarged mesenteric lymph nodes, inflammatory mesenteric fat, and bowel motility. As noted, there is a move towards the use of validated activity scores in clinical practice to improve objectivity in treatment response assessment.

## Complications

### Penetrating disease

**Fig. 4:**
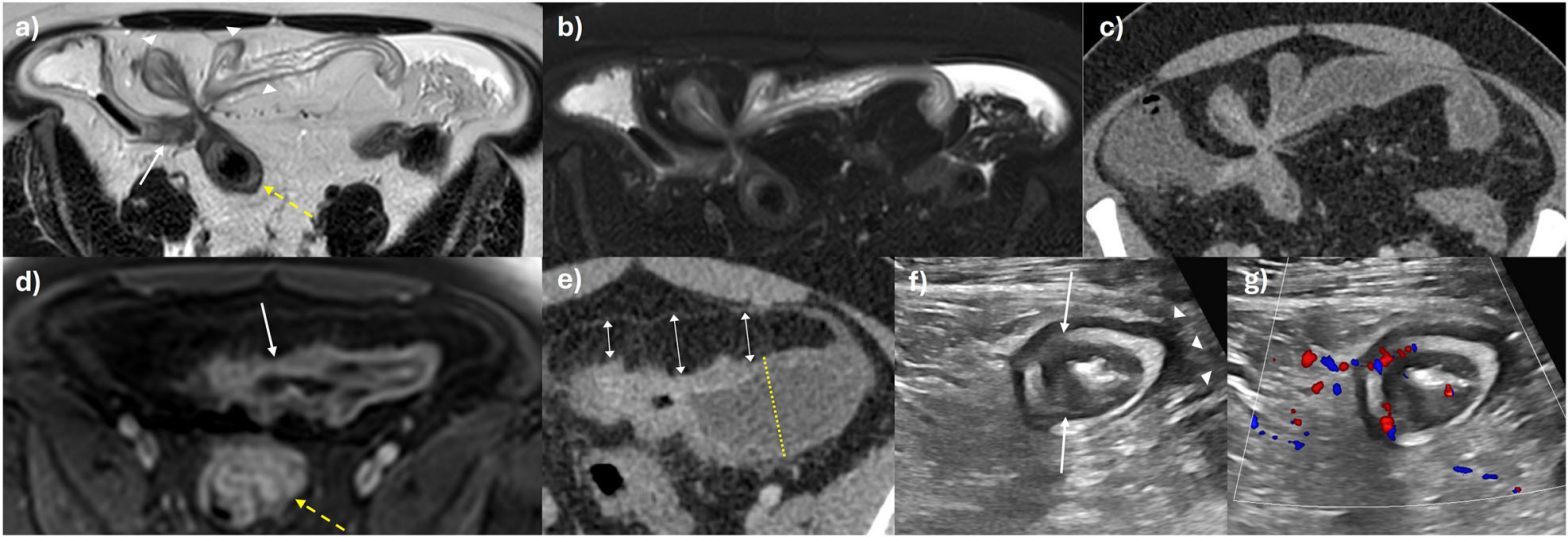
Active disease with penetrating and stricturing complications in a 36-year-old male patient with worsening symptoms, blood, and stool inflammatory markers. **a** Axial T2-weighted and (**b**) axial T2-weighted fat-suppressed images showing complex, fistulating disease of multiple terminal (arrow) and distal ileal loops (arrowheads), and also sigmoid colon (dashed arrow) in a ‘starfish’ configuration. There is severe activity with mural oedema matching the signal intensity of free fluid and perimural oedema, including free fluid. **c** Findings are shown on a CT study with intravenous contrast performed one day prior to MRE. **d** Axial post-contrast T1-weighted image shows severe active disease in the distal ileum upstream of the fistula (arrow) and also the distal sigmoid colon (dashed arrow) with layered transmural hyperenhancement. **e** Persistent narrowing of this same distal ileal segment on CT with mural hyperenhancement and new associated obstruction due to mixed stricturing, active disease (dashed line). There is mesenteric fat wrapping as a sign of chronic disease (arrows). **f** US performed one week later shows an active skip lesion in a mid-ileal loop with mural thickening, patchy hypoechoic submucosal changes, and loss of mural stratification (arrows). The mesenteric border is blurred (arrowheads). **g** There is a corresponding increased colour Doppler signal in these inflamed areas. Patchy mixed hyper- and hypoechoic changes in the mesentery indicated active disease on a background of chronic disease

Penetrating disease in the form of sinuses, fistulae, and/or abscesses can arise when there is severe, uncontrolled transmural inflammation. It is important to describe if there is a sinus (deep transmural ulceration with a linear blind-ending track) or a true formed fistula, i.e., a connection between two epithelialised structures. Fistulae commonly occur between bowel loops, e.g., enterocolic, enteroenteric, especially in the ileum, but can also involve almost any intra-abdominal or pelvic organ, e.g., the urinary bladder, ureters, adnexa, and/or musculature, including the abdominal wall (Figs. 2 and 4) [23]. Patent fistulae may demonstrate passage of intraluminal oral contrast on MRE, and those involving multiple bowel loops often have a “starfish” or stellate configuration.

Intravenous contrast, although not an essential standard MRE sequence, can be useful in penetrating CD, and has particular utility in known or suspected penetrating disease to distinguish abscesses, which may require intervention, from an inflammatory pseudo-mass [24].

### Stricturing disease

**Fig. 5:**
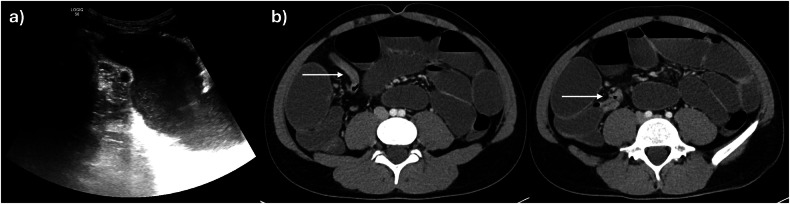
Twenty-six-year-old male patient with obstructive symptoms presented at the emergency department. **a** IUS shows dilated, fluid-filled small bowel loops. **b** Axial emergency contrast-enhanced CT images in portal venous phase confirm small bowel obstruction due to stricturing disease at the level of the terminal ileum (arrows)

**Fig. 6:**
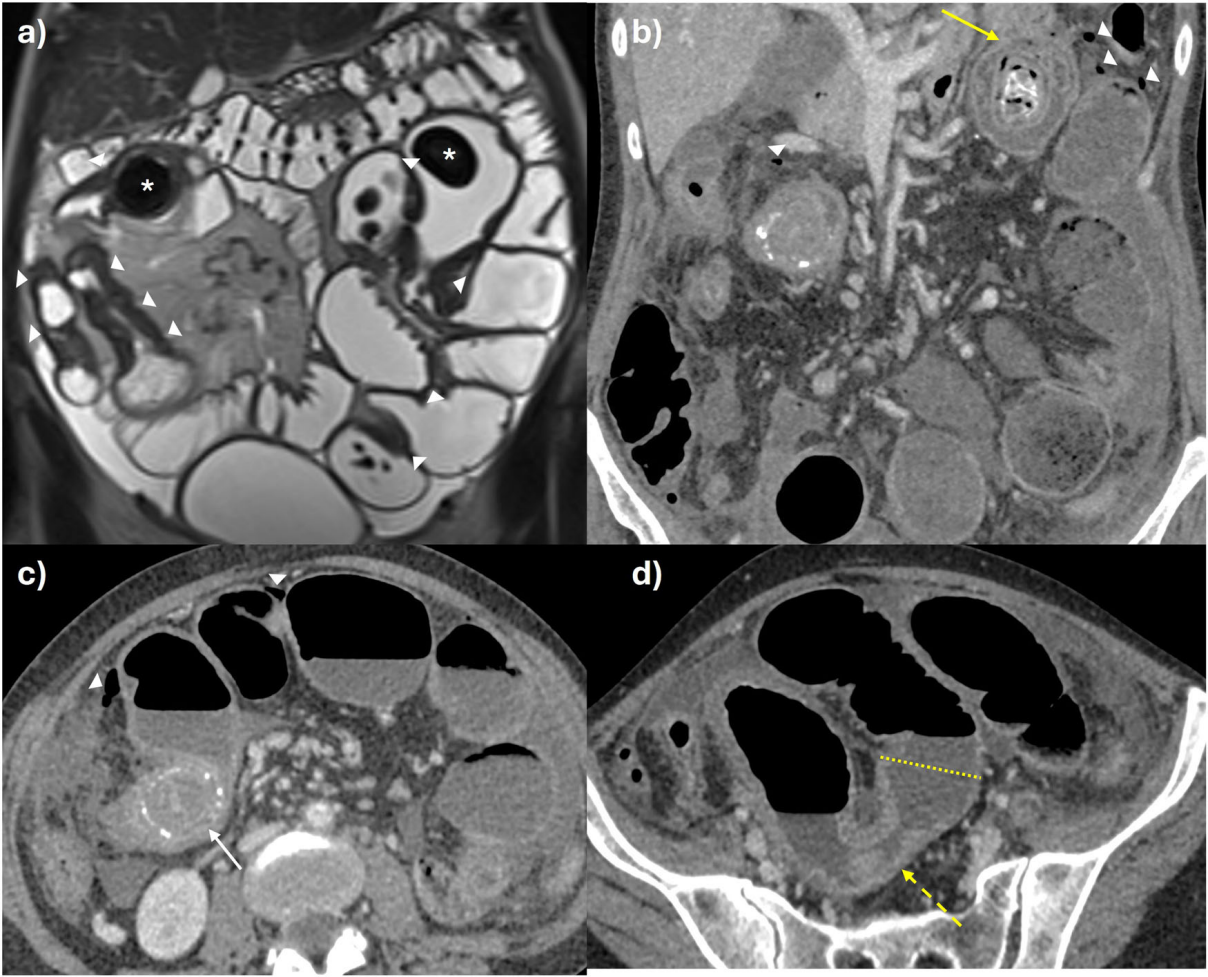
53-year-old female patient with chronic abdominal pain. **a** Coronal T2-weighted MR Enterography image shows multifocal stricturing disease involving most of the small bowel. Strictures (arrowheads) are mostly long (> 3 cm), and there is associated bowel dilation and multiple enteroliths (asterisk on larger examples), indicating chronic obstruction. The strictures are mainly chronic; mostly low T2 signal, maintained on the fat-suppressed sequences, not shown. There is mild superadded activity as shown by the mesenteric free fluid. CT study 2 months later when the patient presented acutely with severe, generalised abdominal pain. **b** Coronal, (**c**) axial images show locules of free gas (arrowheads) and increased free fluid due to perforation from one of the proximal tight strictures with impacted enteroliths (arrow). **d** Axial image shows a deep pelvic stricture (dashed arrow) with mural thickening and hyperenhancement, and dilated bowel loops

Various expert consensus groups have recommended specific criteria to define strictures (i) bowel mural thickening (> 25% for MRE and computed tomography enterography (CTE), > 3 mm for IUS), (ii) fixed luminal narrowing of > 50% relative to normal adjacent bowel loops, and (iii) pre-stenotic dilation (> 3 cm for MRE and CTE, > 2.5 cm for IUS with typically no oral contrast to distend the bowel and so a lower diameter threshold) [25–27]. Strictures in CD have complex histological features, including fibrosis and smooth muscle hypertrophy, and in many cases, there is also superadded acute inflammation. MRE, IUS, and CTE are accurate in stricture detection [25], and CT without luminal contrast is an option in patients with clinically suspected acute bowel obstruction (Fig. 3–6). It has been suggested that fixed luminal narrowing in the absence of proximal dilation is best reported as ‘probable’ stricturing [7].

The stricture length should be reported; if > 5 cm, surgical resection or stricturoplasty is preferred over endoscopic dilation [28]. This factor, along with stricture mural thickness and proximal bowel dilation, is linked to stricture severity [29]. Any coexistent active disease should be flagged, as this may be treated before more invasive interventions.

## Current challenges

This judgement of treatment response can be challenging as active disease invariably coexists with chronic disease within the same segment, with the latter challenging to directly quantify using conventional imaging [25, 30]. In practice, chronic disease such as fibrosis and muscle hypertrophy is inferred by the absence of signs of active inflammation. The radiologist should describe the predominant disease phenotype; active or chronic, or stricturing (often termed “fibrostenotic”), as each has distinct clinical management. It is important to note if there are features of both active and chronic disease, as this may influence the therapeutic approach.

Comparing imaging modalities in the same patient can be difficult. There is an overlap in the signs of disease activity on MRE and IUS, but limited evidence on how best to compare these various signs between modalities. In clinical practice, MRE and IUS are often used interchangeably with considerable variation in modality selection and/or availability. For example, if an MRE shows active disease, treatment response assessment may be performed with IUS. The best option is to select the optimal follow-up imaging modality based on disease location and phenotype and patient tolerance, even if different from the original baseline modality (see ‘Imaging selection for different patient groups’).

## Clinical integration of imaging into patient management

### What does the multidisciplinary team want to know?

**Fig. 7:**
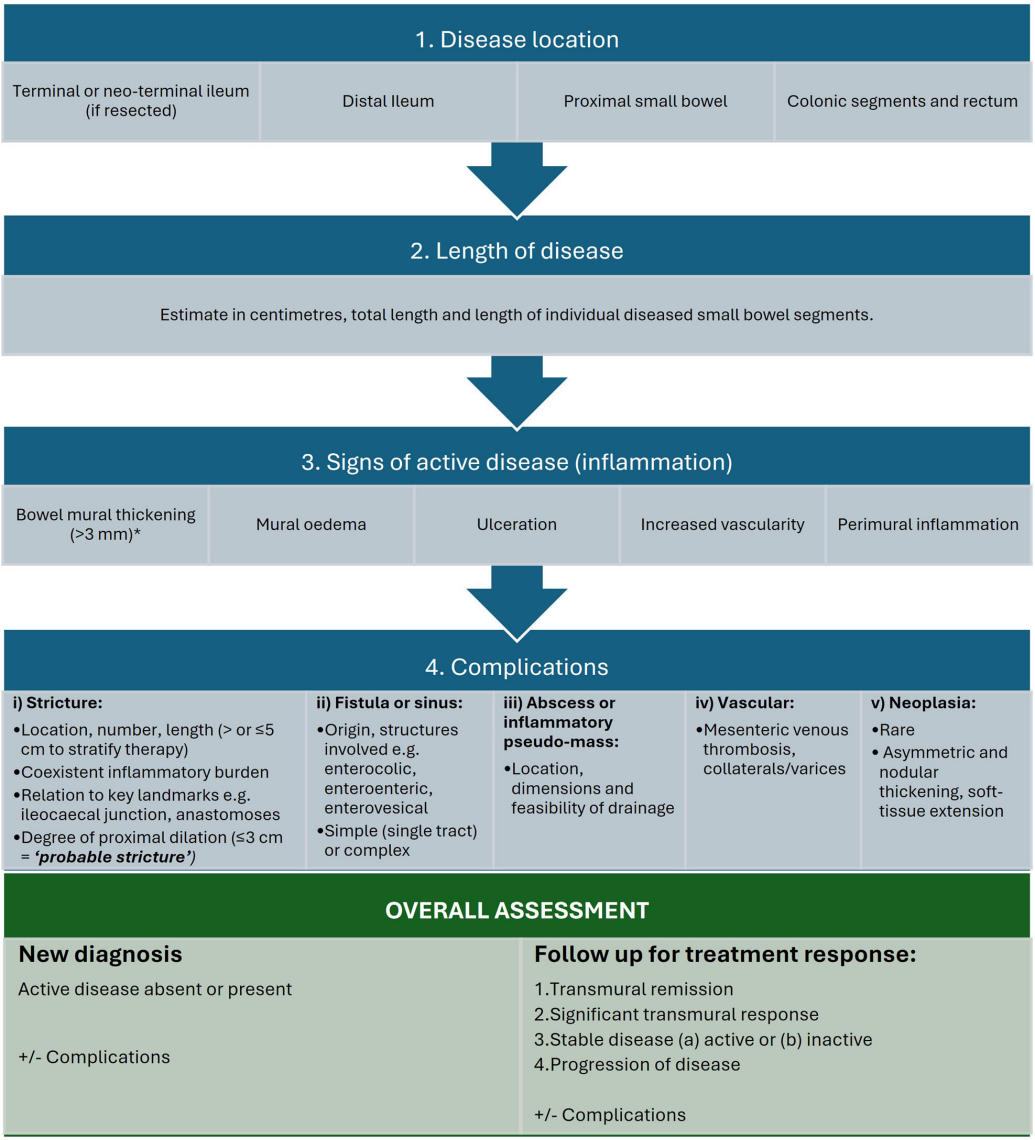
Optimal content and standardised terminology for a structured report in the CD new diagnosis and follow-up settings, based on ECCO-ESGAR recommendations. ^*^Bowel mural thickening can be due to chronic fibrosis, and so other activity signs should be considered

Close interdisciplinary working is key in CD, and the radiologist plays a central role, whether in a specialist or acute setting. To facilitate effective communication, ECCO-ESGAR has recommendations on how to optimise and standardise reporting (Fig. 7) [7].

### Imaging selection for different patient groups

**Fig. 8:**
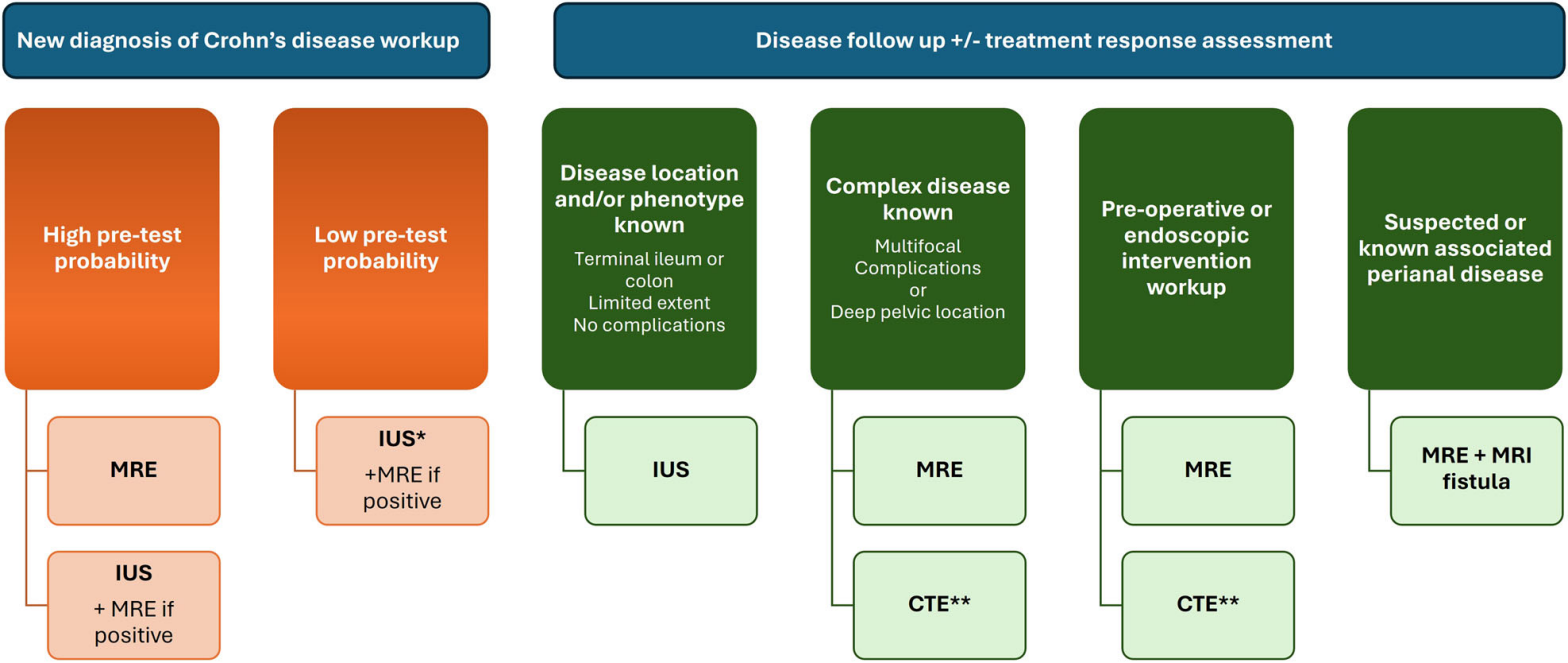
Flowchart illustrating optimal imaging modality selection for distinct clinical scenarios and patient groups. MRE, MR enterography; IUS, intestinal ultrasound; CTE, CT Enterography. ^*^If local IUS capacity allows, otherwise MRE, ^**^CTE is usually reserved as a second-line option with MRE preferred in these clinical scenarios

Appropriate imaging selection is dependent on disease factors (phenotype and anatomical location), individual patient factors (e.g., claustrophobia, patient tolerance and preference, and body mass index), the clinical question, and availability of imaging options delivered by a trained workforce (Fig. 8).

IUS is highly sensitive for terminal ileal and colonic disease in suitable patients, and outperforms MRE for colonic disease assessment, at least for new diagnosis, but also likely for suspected relapse cases [6]. The increased patient acceptability of IUS with its reduced scan duration, no requirement for oral contrast, and ability to communicate with the operator, makes it an excellent choice for follow-up where disease distribution is known [18]. IUS can troubleshoot equivocal MRE findings, such as targeted assessment of bowel loops with graded compression and direct visualisation of peristalsis. There is data that IUS can be a cost and clinically effective first-line test in patients with low probability of CD, but it is resource-intensive, and MRE remains an alternative if there is better capacity [31, 32].

MRE is better suited for initial disease staging at diagnosis; assessing complex, multifocal disease, particularly in suspected disease relapse [6]; deep pelvic location of disease, defining strictures, and mapping disease prior to intervention. MRE is also preferred if there is a clinical suspicion of penetrating disease or abscess, although IUS is also a reasonable first-line alternative.

CT is preferred in the acute setting if there is suspicion of free perforation due to severe penetrating disease, or obstruction where oral contrast would not be tolerated, and/or MRE or CTE would result in diagnostic delay.

Local imaging referral pathways should account for these relative advantages to facilitate appropriate management.

## Summary statement

Collaborative multidisciplinary working is crucial for optimal management of luminal CD. Radiologists are essential in making the initial new diagnosis, and thereafter for appropriate patient follow-up to guide effective and timely management. Early diagnosis is vital to enable early medical therapy and avoid irreversible bowel damage.

MR Enterography and IUS are the recommended first-line cross-sectional imaging modalities, with a clear advantage over colonoscopy in being able to accurately assess the full thickness of the bowel wall, and form the cornerstone of management. CT has a more limited role, selected if MRE and IUS are unavailable, or in the acute setting.

A key objective for radiologists is to phenotype disease by identifying signs of active inflammation, evaluating the relative burden of inflammation and chronic disease, identifying complications, and assessing treatment response. ESGAR recommends the use of standardised terminology and structured reporting to clearly communicate findings and maximise the clinical impact of the report. Radiologists can also guide optimal imaging selection based on local availability, the clinical question, known disease phenotype and location, and other patient-related factors.

## Patient summary

Imaging tests are fundamental to CD management, with radiologists serving an integral part of the clinical care team. MR Enterography, a type of MRI scan, and ultrasound are less invasive methods than repeated endoscopy to accurately assess the disease.

Imaging tests are used when making a new diagnosis of CD, during follow-up to decide how well the disease is responding to medication, and if there are any suspected complications. Imaging is also used to investigate worsening symptoms. Test selection is based on the clinical question, location, and type of disease, local training and services, and patient preference.

## Abbreviations

CTE: Computed tomography enterography
DWI: Diffusion-weighted imaging
ECCO: European Crohn’s and Colitis Organisation
ESGAR: European Society of Gastrointestinal and Abdominal Radiology
IUS: Intestinal Ultrasound
MRE: Magnetic Resonance Enterography
